# Enhanced Performance of the Optimized Dye CF583R in *Direct* Stochastic Optical Reconstruction Microscopy of Active Zones in *Drosophila Melanogaster*

**DOI:** 10.3390/cells13171445

**Published:** 2024-08-28

**Authors:** Marvin Noß, Dmitrij Ljaschenko, Achmed Mrestani

**Affiliations:** 1 Rudolf Schönheimer Institute of Biochemistry, Division of General Biochemistry, Medical Faculty, Leipzig University, D-04103 Leipzig, Germany; 2 Department of Neurology, Leipzig University Medical Center, D-04103 Leipzig, Germany

**Keywords:** super-resolution microscopy, localization microscopy, *direct* stochastic optical reconstruction microscopy, *d*STORM, active zone, Bruchpilot, Brp, hierarchical density-based spatial clustering of applications with noise, HDBSCAN

## Abstract

Super-resolution single-molecule localization microscopy (SMLM) of presynaptic active zones (AZs) and postsynaptic densities contributed to the observation of protein nanoclusters that are involved in defining functional characteristics and in plasticity of synaptic connections. Among SMLM techniques, *direct* stochastic optical reconstruction microscopy (*d*STORM) depends on organic fluorophores that exert high brightness and reliable photoswitching. While multicolor imaging is highly desirable, the requirements necessary for high-quality *d*STORM make it challenging to identify combinations of equally performing, spectrally separated dyes. Red-excited carbocyanine dyes, e.g., Alexa Fluor 647 (AF647) or Cy5, are currently regarded as “gold standard” fluorophores for *d*STORM imaging. However, a recent study introduced a set of chemically modified rhodamine dyes, including CF583R, that promise to display similar performance in *d*STORM. In this study, we defined CF583R’s performance compared to AF647 and CF568 based on a nanoscopic analysis of Bruchpilot (Brp), a nanotopologically well-characterized scaffold protein at *Drosophila melanogaster* AZs. We demonstrate equal suitability of AF647, CF568 and CF583R for basal AZ morphometry, while in Brp subcluster analysis CF583R outperforms CF568 and is on par with AF647. Thus, the AF647/CF583R combination will be useful in future *d*STORM-based analyses of AZs and other subcellularly located marker molecules and their role in physiological and pathophysiological contexts.

## 1. Introduction

Super-resolution light microscopy, i.e., stimulated emission depletion (STED) and variants of SMLM techniques, facilitated imaging studies in multiple areas of neuroscience [1]. The common principle behind SMLM procedures is the determination of spatial coordinates from point spread functions (PSFs) of fluorophores that are attached to molecules of interest [2]. The therefor required separation of PSFs in time is in the case of the SMLM variant *d*STORM (*direct* stochastic optical reconstruction microscopy) achieved by the ability of certain organic dyes to be switched between emitting bright and non-emitting dark states during high-intensity laser excitation (in the order of several kW per cm^2^), usually in the presence of a reducing thiol-containing buffer and, optionally, an oxygen-depleting enzyme mix [3,4]. However, the imaging quality achieved differs substantially between different fluorophores, with the red-excited carbocyanine dyes AF647 and Cy5 dominating over other options [5]. Nevertheless, in most applications, at least two-color super-resolution imaging is highly desirable. Whereas several approaches are available that depend on the use of spectrally similar dyes, including spectral demixing or sequential imaging, oftentimes conventional application of spectrally separated fluorophores is easier to implement.

A recent study introduced a set of chemically modified green-excited rhodamine dyes (CF583R and CF597R) where the rhodamine core benzene was replaced by a positively charged 1,3-disubstituted imidazolium, making the dye more receptive for electron capture, thus stabilizing its dark state and facilitating photoswitching [6].

In the present study, we asked whether the use of CF583R may benefit future *d*STORM-based imaging studies of AZs at the *Drosophila melanogaster* neuromuscular junction (NMJ). Previously, we and others investigated the nanotopology of the abundant AZ scaffold protein Brp [7,8]. Brp, the *Drosophila* ortholog of the mammalian core AZ component ELKS/CAST [9], is crucial for synchronized synaptic release and correct localization of voltage-gated calcium channels (VGCCs) [10,11]. In previous studies, correlation between nanotopological rearrangements of Brp and different functional states of synaptic transmission as well as homeostatic plasticity at the NMJ could be demonstrated, capitalizing on a well-characterized monoclonal mouse antibody against a defined C-terminal epitope (Brp^Nc82^) in combination with Cy5 or AF647 delivered via indirect immunofluorescence [7,8]. Therefore, the Brp^Nc82^ epitope appears to be ideal for benchmarking the performance of newly available *d*STORM dyes in AZ nanoscopy. In this study, we demonstrate that AF647, CF568 and CF583R are equivalent in basal AZ morphometry, while CF583R performs better than CF568 and matches AF647 in more detailed analyses of AZ substructures, making it a promising SMLM tool for future studies.

## 2. Materials and Methods

### 2.1. Fly Husbandry

*Drosophila melanogaster* were raised on a standard cornmeal and molasses medium at 25 °C. Male 3rd instar larvae of strain *w1118* (Bloomington Drosophila Stock Center) were used for experiments.

### 2.2. Dissection, Fixation and Staining

For imaging of NMJs larvae were dissected in ice-cold hemolymph-like solution (HL-3) [12]. Body wall tissue was incubated in 4% paraformaldehyde dissolved in phosphate buffered saline (PBS) for 10 min at room temperature (RT) for chemical fixation. Blocking was performed for 30 min at RT with 5% normal goat serum (Dianova) in PBT (PBS containing 0.05% Triton X-100, Sigma-Aldrich, St. Louis, MO, USA). All staining solutions containing antibodies were also prepared in blocking solution. Incubation with the primary monoclonal mouse antibody Brp^Nc82^ (1:100; AB_2314866, Developmental Studies Hybridoma Bank, Iowa City, IA, USA) was performed for ~16 h overnight at 4 °C. After two short and three 20-min-long washing steps with PBT, secondary antibodies were applied for 3 h at RT at the following concentrations: Goat α-mouse F(ab’)_2_ coupled Alexa Fluor 647 (AF647, 1:1000, degree of labeling (DOL) 3 (LOT 2486562); A-21237, Thermofisher, Waltham, MA, USA), goat α-mouse IgG coupled CF568 (1:500, DOL 1; 20800-500 uL, Biotium, Fremont, CA, USA), goat α-mouse IgG coupled CF583R (1:500, DOL 1; 20792-500 uL, Biotium). Secondary staining was followed by two short and three 20-min-long washing steps with PBT. Preparations were kept in PBS at 4 °C until imaging. Data were exclusively acquired from NMJs formed at abdominal muscles 6 and 7 in segments A2 to A4.

### 2.3. dSTORM (Direct Stochastic Optical Reconstruction Micrsocopy)

Preparations were embedded in a standard photoswitching buffer [4] containing 200 mM cysteamine hydrochloride (M6500, Sigma-Aldrich) in PBS with pH adjusted to ~7.8. Probes were covered with high-precision borosilicate glass slides (thickness 0.17 mm; LH25.2, Roth, Karlsruhe, Germany). Images were acquired at ~21 °C room temperature using a custom-built *d*STORM setup that was based on a pneumatic vibration isolation workstation and assembled around an Olympus IX70-S1F2 inverted microscope equipped with an oil immersion objective (60× magnification, NA 1.5; UPLAPO60XOHR, Olympus, Tokio, Japan). An objective-type total internal reflection fluorescence (TIRF) configuration was implemented, and images were acquired applying highly inclined and laminated optical sheet (HILO) microscopy [13]. 640 nm (200 mW; IBEAM-SMART-640-S-HP, TOPTICA, Gräfelfing, Germany) and 561 nm (300 mW; Sapphire 561-300 LPX CDRH, Coherent, Santa Clara, CA, USA) lasers were used for excitation of AF647 and CF568 or CF583R, respectively. Lasers were combined by a dichroic mirror (LaserMUX 561-594R, F38-M05, AHF Analysentechnik, Tübingen, Germany) and reflected onto the probe via an excitation dichroic mirror (Quad Line beamsplitter zt405/488/561/640rpc flat, F73-410, AHF Analysentechnik) after passing through a multiband clean-up filter (Quad Line laser clean-up ZET405/488/561/640x, F59-405, AHF Analysentechnik). Both lasers were operated at 150 mW output power, and additional lenses were used to illuminate a region of ~20 × 20 µm^2^. Emitted fluorescence passed through an additional multiband filter (Quad Line rejection band ZET405/488/561/640m, F57-405, AHF Analysentechnik) and was spectrally separated with an OptoSplit II beamsplitter (internal magnification ~1.68×; Cairn Research, Faversham, UK), equipped with a dichroic mirror (ZT633rdc, upgraded to ultraflat and 2 mm thickness ZT-UF2, Chroma, Bellows Falls, VT, USA), and divided onto different regions of the chip of a single camera (iXon Life 888 EMCDD, Andor, Belfast, Northern Ireland). We collected 15,000 frames with an exposure time of 12 ms per super-resolved image at an EM gain of 300. Objective and OptoSplit II magnification resulted in a final pixel size of 123 nm for both channels. Fitting of PSFs and generation of localization tables was performed using the open source rapi*d*STORM software [14]. Only localizations with an A/D count of minimum 3000 and 1000 for AF647 and CF568 or CF583R, respectively, were included in further analyses. Localization precision was determined with the NeNa algorithm (nearest neighbor-based analysis) [15], implemented in the LAMA software package (LocAlization Microscopy Analyzer) [16].

### 2.4. Data Analysis

Analysis of localization data was performed essentially as previously described [8,17,18,19] using custom-written Python code (language version 3.10.5) and the Python interface Jupyter [20]. Regions of interest (ROIs) corresponding to maximum 6 terminal type Ib boutons were marked in reconstructed binned images (pixel size 10 nm) from rapi*d*STORM using FIJI [21]. Brightness (camera A/D count) was determined as mean per image for the localizations inside ROIs. Clusters of Brp^Nc82^ localizations, i.e., AZs, were extracted using the Python implementation of “hierarchical density-based spatial clustering of applications with noise” (HDBSCAN) [22]. We tested the influence of several settings of the main free parameters and all combinations thereof (“minimum cluster size”: 10–90 in increments of 10 and 100–500 in increments of 100; “minimum samples”: 2, 5–25 in increments of 5 and 30–100 in increments of 10) on the number of detected AZs per image. The final analysis parameters for all three dyes (minimum cluster size = 100, minimum samples = 25) were set in a stable range that yielded most plausible clustering results. AZ areas were determined by computing 2D alpha shapes in Python CGAL (Computational Geometry Algorithms Library). Expectedly, increasing alpha values yielded increasingly larger AZ areas (tested parameters: alpha = x^2^ nm^2^ with x ranging from 5 to 200 in increments of 5). The final quantification was performed with the first alpha parameter that yielded a percentage AZ area increase per step below 5%. Exclusion criteria for the ultimately presented AZs were area ≤ 0.03 μm^2^ and ≥0.3 μm^2^ [7,8]. H functions were computed using the Python package Astropy [23] without correction for edge effects, and the functions were averaged for all individual AZs per dye before determining maxima as indicators of the substructures’ radii [24]. A second-level HDBSCAN was performed with parameters adjusted individually for the different dyes to yield Brp subclusters (SCs) with radii matching the H functions’ maxima [8]. SC areas were computed with alpha = 300 nm^2^ for all conditions.

### 2.5. Statistics

Statistical evaluations were performed with Sigma Plot 13 (Systat Software, Erkrath, Germany). Data are presented as median (25th–75th percentile) unless indicated otherwise. Shapiro–Wilk was used to test normality. Since the statistically compared data sets were not normally distributed, one-way ANOVA on ranks was performed, including pairwise comparisons using Dunn’s method. Numerical values are reported in text, sample sizes (n) in text and figure legends and *p*-values in text and figures. Statistically significant difference was assumed if *p* < 0.05. Plots were generated with Sigma Plot or Python library Matplotlib.

## 3. Results

### 3.1. Application of AF647, CF568 and CF583R Permits dSTORM Imaging of AZs

**Figure 1:**
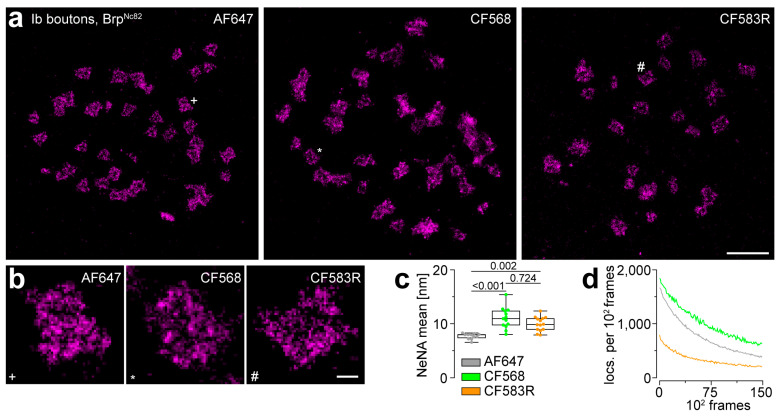
Application of Alexa Fluor 647, CF568 and CF583R for *direct* stochastic optical reconstruction microscopy of Bruchpilot. (**a**) Binned images (10 nm pixels) of *direct* stochastic optical reconstruction microscopy (*d*STORM) of Bruchpilot (Brp) stained with the primary monoclonal mouse antibody Brp^Nc82^ and secondary F(ab’)_2_ fragments coupled to Alexa Fluor 647 (AF647, **left**) or secondary IgGs, either coupled to CF568 (**middle**) or CF583R (**right**), at distal type Ib boutons of the 3rd instar larval *Drosophila melanogaster* neuromuscular junction (NMJ) formed at abdominal muscles 6 and 7. Symbols (+, *, #) mark the enlarged individual active zones (AZs) in (**b**). (**b**) Enlarged AZs from (**a**). (**c**) Localization precision determined by NeNA algorithm (nearest neighbor-based analysis, see Materials and Methods) for AF647 (grey, n = 11 images from 3 animals), CF568 (green, n = 13 images from 4 animals) and CF583R (orange, n = 14 images from 4 animals) shown as boxplots, where horizontal lines represent medians, boxes quartiles and whiskers minimum and maximum values, combined with swarm plots displaying individual data points. (**d**) Number of *d*STORM localizations (locs.) per 100 frames for the three different dyes (averaged for the same images as in (**c**)). Scale bars in (**a**) 1 µm, in (**b**) 100 nm.

To evaluate the performance of CF583R in *d*STORM-based AZ nanoscopy at the *Drosophila* NMJ and compare it to the red-emitting “gold standard” dye AF647 and the “next best” green-excited dye CF568 [6], we assembled a *d*STORM setup equipped with 561 nm and 640 nm excitation laser lines. In favor of a low complexity, easily applicable and reproducible experimental setup, we omitted an UV spectrum activation laser and embedded the specimen in standard pH-adjusted PBS buffer, which contained cysteamine without an oxygen scavenger enzyme mix. Imaging of the AZ scaffold protein Brp yielded comparable results for the three different dyes, with easily separable AZs (Figure 1a) and clearly visible substructures within individual AZs (Figure 1b), as known from earlier *d*STORM imaging of Brp using AF647 [7,8].

To assess data quality, we calculated the localization precision achieved by the different dyes using the NeNA algorithm (nearest neighbor-based analysis) [15]. As expected, AF647 with 8 (7–8) nm (median (25th–75th percentile)) delivered a significantly better localization precision compared to CF568 with 11 (10–12) nm (Figure 1c; AF647: n = 11 images from 3 animals, CF568: n = 13 images from 4 animals, one-way ANOVA on ranks *p* < 0.001, pairwise comparison AF647 vs. CF568 *p* < 0.001). AF647 localization precision was also significantly better compared to CF583R with 10 (9–11) nm (n = 14 from 4 animals, *p* = 0.002), though the difference was smaller in this case. The slight numerical difference between the two rhodamine dyes was not statistically significant (*p* = 0.724). The differences in localization precision may be largely due to the distinct brightness of the fluorophores (camera A/D count AF647: 8228 (7616–8827), CF568: 4144 (3758–5318), CF583R: 3879 (3297–4380), one-way ANOVA on ranks *p* < 0.001, pairwise comparison AF647 vs. CF568 *p* < 0.001, AF647 vs. CF583R *p* < 0.001, CF568 vs. CF583R *p* = 1.0). In summary, AF647 outclasses CF568 and CF583R in terms of localization precision, while CF583R may perform slightly better than CF568. In the next step, we counted Brp^Nc82^ localizations captured per 100 camera frames for the different dyes (Figure 1d). Most localizations were detected using CF568, followed by AF647, while least localizations were captured using CF583R. Detection of fewer localizations may be caused by multiple factors, e.g., lower labeling density and less stability to bleaching, but also, as has been previously demonstrated for CF583R [6], greater preference of the non-emitting dark state, i.e., better suitability for photoswitching.

### 3.2. AF647, CF568 and CF583R Perform Equally in Basal AZ Morphometry

**Figure 2:**
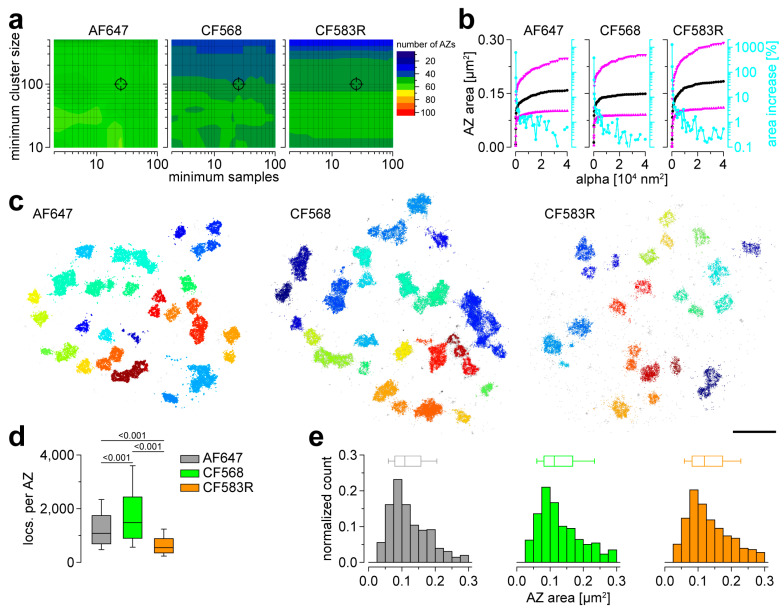
AF647, CF568 and CF583R yield sufficient localization to obtain basal AZ morphometric parameters. (**a**) Contour plots of the median number of AZs per *d*STORM image depending on the HDBSCAN (hierarchical density-based spatial clustering of applications with noise) parameters “minimum samples” and “minimum cluster size” for AF647 (left, n = 11 images from 3 animals), CF568 (middle, n = 13 images from 4 animals) and CF583R (right, n = 14 images from 4 animals). Crosshairs indicate the parameter combination used in (**b**–**e**) (25 and 100, respectively, for all three conditions). (**b**) Line and scatter plots of median AZ area (black) as well as 25th and 75th percentiles (up- and downward magenta triangles, respectively, same images as in (**a**)) plotted against alpha values used for determination of alpha shape areas. Blue line and scatter plots indicate the percent increases of AZ areas with increasing alpha (zeros omitted due to logarithmic scale). The relative increase dropped below 5% at alpha values of 1225, 900 and 1600 nm^2^ for AF647, CF568 and CF583R, respectively (the selected parameters used for quantification in (**e**)). (**c**) Scatter plots of *d*STORM localizations of type Ib boutons shown in Figure 1a. Different colors indicate the identity of AZs detected by HDBSCAN, grey dots are unclustered localizations. (**d**) Numbers of localizations per AZ for AF647 (grey, 557 AZs from 11 NMJs and 3 animals), CF568 (green, 514 AZs from 13 NMJs and 4 animals) and CF583R (orange, 607 AZs from 14 NMJs and 4 animals) shown as boxplots, where horizontal lines represent medians, boxes quartiles and whiskers 10th and 90th percentiles. (**e**) Area of individual AZs using the three different dyes shown as histograms and boxplots (same AZs as in (**d**)). Scale bar in (**c**) 1 µm.

After having established Brp imaging applying three different *d*STORM-ready dyes, we turned to quantitative AZ morphometry in large data sets. To this effect, we used a previously introduced analysis pipeline based on HDBSCAN [8]. First, we explored optimal clustering parameters evaluating the number of detected AZs per image over a wide range of varying free parameters “minimum samples” and “minimum cluster size” (Figure 2a). Interestingly, robustness of clustering results for all three dyes permitted application of the same parameter combination used in earlier nanoscopic analyses of Brp using AF647 [8] or AF532 [17,18]. Next, we applied an analysis routine introduced in earlier research to determine optimal parameters for AZ area determination using alpha shapes [19] and received robust results for AF647, CF568 and CF583R (Figure 2b). Visualization of the results applying the optimized parameters delivered convincing AZ segmentation for all three imaging conditions (Figure 2c). After having established a robust quantification routine, we analyzed the number of Brp^Nc82^ localizations (locs.) per individual AZ and found that CF568 AZs contained the highest number, whereas CF583R AZs had fewer locs. compared to both other dyes (Figure 2d; AF647: 1085 (695–1742), CF568: 1481 (901–2436) and CF583R: 547 (356–887) locs. per AZ, one-way ANOVA on ranks *p* < 0.001, pairwise comparison AF647 vs. CF568, AF647 vs. CF583R and CF568 vs. CF583R *p* < 0.001, respectively, n = 557 AZs from 11 NMJs and 3 animals, 514 AZs from 13 NMJs and 4 animals and 607 AZs from 14 NMJs and 4 animals for AF647, CF568 and CF583R, respectively). In the next step, we analyzed the distributions of AZ areas for all three dyes (Figure 2e). Strikingly, we obtained similar histograms for all conditions (AF647: 0.109 (0.079–0.157), CF568: 0.112 (0.081–0.168), CF583R: 0.119 (0.082–0.174) µm^2^, one-way ANOVA on ranks *p* = 0.021, pairwise comparison AF647 vs. CF568 *p* = 0.297, AF647 vs. CF583R *p* = 0.017, CF568 vs. CF583R *p* = 0.921; note that AZ areas were computed using individual alpha values for alpha shape determination for each dye). This demonstrates robust performance of AF647, CF568 and CF583R in basal AZ morphometry using *d*STORM, with results for the Brp^Nc82^ epitope that are in line with previous analyses [7,8].

### 3.3. CF583R Outperforms CF568 and Matches AF647 in AZ Nanocluster Analyses

**Figure 3:**
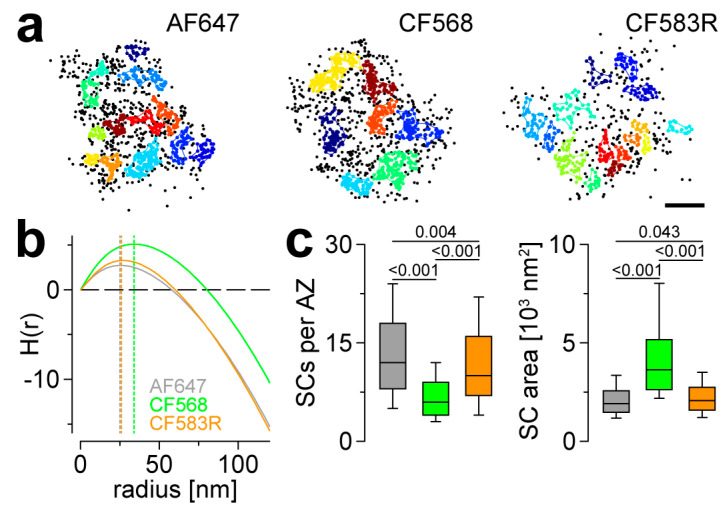
CF583R and AF647 perform equally in AZ nanocluster analyses. (**a**) Same AZs as in Figure 1b shown as scatter plots of *d*STORM localizations. Brp subclusters (SCs) per AZ were detected by a second-level HDBSCAN on individual AZs with parameters individually adjusted for each imaging condition to yield comparable SC radii with respect to H function maxima in (**b**). Parameters for minimum cluster size and minimum samples were 22 and 5, 44 and 11 as well as 15 and 3 for AF647, CF568 and CF583R, respectively. SC identity is indicated by different colors, black dots are unclustered localizations and colored lines show alpha shapes used for area determination in (**c**). (**b**) Averaged H functions (as derivatives of Ripley’s K function) for AF647 (grey), CF568 (green) and CF583R (orange) shown as straight lines. Colored dashed lines indicate H function maxima (AF647: 25 nm, CF568, 34 nm, CF583R: 26 nm, n = 535, 514 and 594 averaged H functions, respectively), while the dashed black line shows the prediction for a random Poisson distribution. (**c**) Number of SCs per AZ (left) and SC area (right) for the three different dyes shown as boxplots (n = 557 AZs from 11 NMJs and 3 animals, 502 AZs from 13 NMJs and 4 animals and 607 AZs from 14 NMJs and 4 animals for AF647, CF568 and CF583R, respectively). Scale bar in (**a**) 100 nm.

Imaging studies relying on super-resolution localization microscopy have contributed to the definition of pre- and postsynaptic protein nanoclusters whose amount and precise spatial arrangement are crucial for determining functional properties of synapses and which display remarkable reorganization during synaptic plasticity (e.g., [7,8,25,26,27,28,29,30]). The nano-arrangement of Brp^Nc82^ at *Drosophila* AZs has been characterized in detail [7,8]. Thus, it represents an ideal epitope to assess the performance of a new experimental conjunction in AZ nanotopology. To this end, we performed previously introduced analyses based on a combination of a second-level HDBSCAN and Ripley’s K function ([8], see Materials and Methods) to extract Brp^Nc82^ subclusters (SCs) using the three different dyes (Figure 3a,b).

HDBSCAN parameters were adjusted to yield results comparable to the H functions’ (derivatives of Ripley’s K function) maxima (Figure 3b). Interestingly, AF647 and CF583R delivered similar results concerning Brp^Nc82^ SC radii with ~25 nm and ~26 nm, respectively, matching earlier results [8] and clearly outperforming CF568 with ~34 nm (Figure 3b). Whereas the number of SCs per AZ was significantly lower and, consecutively, the SC area was significantly higher using both CF568 and CF583R compared to AF647, the differences were obviously more dramatic in CF568 AZs (Figure 3c; SCs per AZ: AF647: 12 (8–18), CF568: 6 (4–9), CF583R: 10 (7–16) SCs per AZ, one-way ANOVA on ranks *p* < 0.001, pairwise comparison AF647 vs. CF568 *p* < 0.001, AF647 vs. CF583R *p* = 0.004, CF568 vs. CF583R *p* < 0.001; SC area: AF647: 1911 (1475–2568), CF568: 3632 (2625–5167), CF583R: 2067 (1587–2750) nm^2^, one-way ANOVA on ranks *p* < 0.001, pairwise comparison AF647 vs. CF568 *p* < 0.001, AF647 vs. CF583R *p* = 0.043, CF568 vs. CF583R *p* < 0.001, n = 557 AZs from 11 NMJs and 3 animals, 502 AZs from 13 NMJs and 4 animals and 607 AZs from 14 NMJs and 4 animals for AF647, CF568 and CF583R, respectively). In summary, the optimized dye CF583R outperformed CF568 in terms of Brp^Nc82^ SC quantification and delivered acceptably similar results compared to AF647.

## 4. Discussion

In this study, we analyzed the suitability of the optimized dye CF583R for *d*STORM imaging of the presynaptic AZ scaffold protein Brp at *Drosophila* NMJs and compared it to AF647 as well as CF568. While the red-excited carbocyanine dye AF647 appears to remain the most favorable *d*STORM fluorophore, CF583R delivered acceptably similar results. In previous nanotopological analyses of the core AZ proteins Unc-13 and RIM [17,18], we relied on the classical rhodamine dye AF532 as a second label for *d*STORM imaging. Thereby, the molecule of interest was imaged in the high-quality AF647 channel, whereas the green channel could be used as reference signal in terms of Brp^Nc82^ as an AZ marker. In our hands, AF532 yielded sufficient data quality for basal morphometry (compare Figure 2); however, analysis of Brp SCs (compare Figure 3) was not possible at an acceptable level. Especially with respect to analyzing AZ substructures, CF583R showed improved performance compared to CF568, making it a promising tool for future two-color *d*STORM applications to clarify the relative positioning between crucial AZ components as well as to VGCCs. Further improvement regarding localization yield should, if necessary, be achievable by incorporation of a UV spectrum activating laser into the setup, at least for the green-excited CF583R channel, as well as using an oxygen scavenger enzyme mix. However, in our hands, the latter option did not induce observable effects in past studies, at least at the level of AZ mesoscale morphometry. In summary, the AF647/CF583R dye pair might be the favorite combination for upcoming *d*STORM-based imaging studies of AZs at *Drosophila* NMJs and, possibly, other preparations.

## Data Availability

All data sets and code can be obtained from the corresponding author without restrictions.
